# Development and Validation of a Prediction Model for Tube Feeding Dependence after Curative (Chemo-) Radiation in Head and Neck Cancer

**DOI:** 10.1371/journal.pone.0094879

**Published:** 2014-04-15

**Authors:** Kim Wopken, Hendrik P. Bijl, Arjen van der Schaaf, Miranda E. Christianen, Olga Chouvalova, Sjoukje F. Oosting, Bernard F. A. M. van der Laan, Jan L. N. Roodenburg, C. René Leemans, Ben J. Slotman, Patricia Doornaert, Roel J. H. M. Steenbakkers, Irma M. Verdonck-de Leeuw, Johannes A. Langendijk

**Affiliations:** 1 Department of Radiation Oncology, University of Groningen, University Medical Center Groningen, Groningen, the Netherlands; 2 Department of Radiation Oncology, VU University Medical Center, Amsterdam, the Netherlands; 3 Department of Medical Oncology, University of Groningen, University Medical Center Groningen, Groningen, the Netherlands; 4 Department of Otolaryngology/Head and Neck Surgery, University of Groningen, University Medical Center Groningen, Groningen, the Netherlands; 5 Department of Oral and Maxillofacial Surgery, University of Groningen, University Medical Center Groningen, Groningen, the Netherlands; 6 Department of Otolaryngology-Head and Neck Surgery, VU University Medical Center, Amsterdam, the Netherlands; University of Michigan, United States of America

## Abstract

**Background:**

Curative radiotherapy or chemoradiation for head and neck cancer (HNC) may result in severe acute and late side effects, including tube feeding dependence.

The purpose of this prospective cohort study was to develop a prediction model for tube feeding dependence 6 months (TUBE_M6_) after curative (chemo-) radiotherapy in HNC patients.

**Patients and Methods:**

Tube feeding dependence was scored prospectively. To develop the multivariable model, a group LASSO analysis was carried out, with TUBE_M6_ as the primary endpoint (n = 427). The model was then validated in a test cohort (n = 183). The training cohort was divided into three groups based on the risk of TUBE_M6_ to test whether the model could be extrapolated to later time points (12, 18 and 24 months).

**Results:**

Most important predictors for TUBE_M6_ were weight loss prior to treatment, advanced T-stage, positive N-stage, bilateral neck irradiation, accelerated radiotherapy and chemoradiation. Model performance was good, with an Area under the Curve of 0.86 in the training cohort and 0.82 in the test cohort. The TUBE_M6_-based risk groups were significantly associated with tube feeding dependence at later time points (p<0.001).

**Conclusion:**

We established an externally validated predictive model for tube feeding dependence after curative radiotherapy or chemoradiation, which can be used to predict TUBE_M6_.

## Introduction

Patients with head and neck cancer (HNC) often receive intensive anticancer treatment such as radiotherapy as single modality or in combination with chemotherapy and/or targeted agents such as cetuximab. Many patients may have severe difficulties maintaining adequate nutritional intake prior to treatment. This is caused by local tumor growth, which leads to swallowing dysfunction, trismus, odynophagia, dysgeusia and aspiration. In addition, anticancer therapy causes severe side effects such as acute mucositis and xerostomia inducing swallowing dysfunction. After completing such therapy, a substantial proportion of patients without baseline swallowing dysfunction ultimately develop persistent or even progressive swallowing dysfunction. In some cases they require tube feeding for a long period of time [1].

Recently it was shown that swallowing dysfunction has a major impact on health-related quality of life [2]. With grade III–IV swallowing dysfunction according to the RTOG Late Radiation Morbidity Scoring System, the most important general dimensions of health-related quality of life were moderately to severely affected. Moreover, swallowing dysfunction has been associated with psychological distress not only in patients themselves, but also in their spouses [3]. These results demonstrate that swallowing dysfunction in general, and tube feeding dependence in particular, are clinically relevant long-term side effects after curative (chemo-) radiotherapy.

Moreover, high-intensity treatment regimens have resulted in improved survival, but with higher rates of tube feeding dependence in these survivors [4], [5]. The prevalence of patients with long-term tube feeding dependence is therefore expected to increase.

Previous studies have shown that the dose to the larynx and pharyngeal musculature in radiotherapy treatment of HNC is associated with the risk of long-term swallowing dysfunction [6]–[8] and are considered swallowing organs at risk. Advanced radiation delivery techniques such as intensity modulated radiotherapy (IMRT) have been used to reduce the radiation dose to the swallowing organs at risk [9]. Promising results have been reported on the use of swallowing exercises before and during treatment to reduce the risk of persisting swallowing dysfunction after curative (chemo-) radiation [10], [11]. Thus, predictive models that can identify patients at increased risk of tube feeding dependence after curative (chemo-) radiotherapy before starting treatment would allow selection of suitable candidates for preventive strategies, such as swallowing sparing IMRT and/or preventive swallowing exercises.

Therefore, the main purpose of this study was to develop a prediction model for tube feeding dependence after curative (chemo-) radiotherapy in HNC based on pretreatment characteristics that can be used to improve selection of patients, prior to treatment, for these preventive measures and/or support decision making with regard to the treatment strategy in an early stage (e.g. definitive radiotherapy versus primary surgery). This prediction model was validated in an external and independent prospective cohort to further support its general applicability.

## Material and Methods

### Ethics statement

All patients were subjected to a prospective data registration program in which complications and treatment results in terms of local control and survival are prospectively assessed. This is done within the framework of routine clinical practice in which outcome and complications are systemically scored as part of a quality assurance program. All data obtained and used for this study has been anonymized.

The (Dutch) Medical Research Involving Human Subjects Act is not applicable to data collection as part of routine clinical practice and use of these data for scientific papers regarding the quality assurance program. Only research that is within the scope of the Medical Research Involving Human Subjects Act needs approval from an (accredited) ethics committee. Therefore, the hospital ethics committee (the Medisch Ethische Toetsingscommissie; METc) concluded that data collection by this program is regarded as part of routine patient care and granted us a waiver from needing ethical approval for the conduct of this study.

In the Netherlands a patient of course has to give his/her consent for the collection of the extra data on behalf of the quality assurance program and the use of these data for scientific papers regarding the quality assurance program. However, according to Dutch legislation, consent is free of form, and verbal consent is sufficient. Therefore, patients were asked to participate in this quality assurance program and asked for permission to use their data for the program and scientific papers regarding the program. Refusal of participation was recorded in their medical record.

### Patients

The population of this prospective cohort study was composed of 610 consecutive patients with carcinoma of the mucosal surfaces of the larynx, oropharynx, oral cavity, hypopharynx and nasopharynx, who received curative radiotherapy with or without chemotherapy or cetuximab. Data from patients treated at our hospital were used to develop the prediction model (training cohort: 427 patients), while data from patients treated at another hospital were used to externally validate the model (test cohort: 183 patients).

Baseline weight loss was defined as the percentage of total body weight lost during the 6 months prior to radiation, with 1 to 10% weight loss defined as moderate and more than 10% defined as severe weight loss.

As we were primarily interested in radiation-induced swallowing dysfunction, patients that used a feeding tube at baseline were excluded from this analysis (RTOG grade 3–4). Moreover, patients had to be free of local recurrence or distant metastases at the time of assessment of swallowing dysfunction.

### Treatment

All patients were treated either with conventional 3D conformal radiotherapy (3D-CRT) or IMRT. The dose to the parotid glands was reduced as much as possible. In the cohorts included in this analysis, no dose constraints for the swallowing organs at risk were used.

Patients undergoing concomitant chemoradiotherapy were treated with conventional fractionation (2.0 Gray (Gy) per fraction, 5 times per week up to 70 Gy in 7 weeks). Patients with stage I–II and stage III–IV tumors who were considered ineligible for (chemo-) radiotherapy were treated with accelerated radiotherapy with a concomitant boost technique (2.0 Gy per fraction, 6 times per week up to 70 Gy in 6 weeks). Since 2008, patients with locally advanced (stage III–IV) tumors, for whom chemotherapy was considered infeasible, have been treated with cetuximab using a loading dose of 400 mg/m^2^ one week prior to radiotherapy and a weekly dose of 250 mg/m^2^ during accelerated radiotherapy (2.0 Gy per fraction, 6 times per week up to 70 Gy in 6 weeks).

In the training cohort, concomitant chemotherapy consisted of cisplatin 100 mg/m^2^ on days 1, 22 and 43. In the test cohort, concomitant chemotherapy consisted of 3 cycles of carboplatin (300–350 mg/m^2^) on day 1 and 5-fluorouracil (5-FU) on days 1 to 4 as a continuous infusion (600 mg/m^2^/24 hours) every 3 weeks.

At both institutions, prophylactic PEG tube placement was standard of care in all patients treated with curative concomitant chemoradiation and patients were instructed to refrain from using the PEG-tube. In patients with significant weight loss (>5% weight loss in 1 month or >10% in 6 months or BMI <18.5 kg/m^2^) and/or low nutritional intake (less than half of daily requirements for energy, proteins or fluids) and/or severe swallowing dysfunction prior to treatment, PEG tubes were placed prior to treatment. However, these patients were excluded from the analysis.

Reactive placement of feeding tubes was used for patients with significant weight loss or swallowing dysfunction during treatment; in this situation a nasogastric feeding tube was placed during treatment if swallowing problems were considered temporarily and expected to recover soon. In case of severe swallowing problems early during treatment and/or expected to sustain for a longer period of time, there was a preference for PEG-tube placement.

### Follow up schedule and assessments

In both hospitals, acute and late radiation-induced side effects were prospectively assessed according to the RTOG/EORTC Acute and Late Radiation Morbidity Scoring System. Tube feeding dependence was scored separately. For the present analysis, the primary endpoint was tube feeding, either with PEG (percutaneous endoscopic gastrostomy) or nasogastric tube at 6 months after completion of treatment (TUBE_M6_). Patients were considered tube feeding dependent if a feeding tube was present and used because oral intake was limited or impossible.

### Definition of risk groups

The total population of the training cohort was divided into three risk groups based on the risk on TUBE_M6_. The division into low, intermediate and high risk groups was arbitrary: patients were considered low risk when the probability for TUBE_M6_ was ≤5%, intermediate risk when this value was >5–15% and high risk for values >15%. To determine whether the model could be extrapolated for the same patients at later time points, the positive and negative predictive values for TUBE_M6_ were calculated at 12, 18 and 24 months.

### Statistics

After the regression analysis, the variance inflation factor was calculated to check for high correlations between candidate prognostic variables. There were no high correlations and, therefore, no changes were made to the variables.

For the development of the prediction model the least absolute shrinkage and selection operator (LASSO) method was used, which is a logistic regression analysis with a bound on the absolute magnitude of the regression coefficients [12]. This method includes all variables in the modeling process but only a subset of predictor variables are eventually included in the model, setting the coefficients of variables that have negligible effects to zero. The LASSO method has been successfully applied to build Normal Tissue Complication (NTCP) models for HNC patients [13]. Given the inclusion of categorical variables in the current data, the group-LASSO (variant of LASSO) was used for building the prediction models. The amount of shrinkage was selected by optimizing the Bayesian information criterion (BIC) over the regularization path.

The environment for statistical computing R (R Development Core Team, R: A language and Environment for statistical Computing, Version 2.15, Vienna, 2012) was used to do the calculation. The package ‘grpreg’ was used to build the group-LASSO model.

For the selected variables x_i_ and their fitted coefficients β_i_, the Normal Tissue Complication Probability (NTCP) is given by:


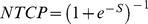
, *in which*

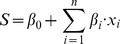



Model performance was described using different validation measures [14], [15]. The discriminating ability of the model was described by the area under the curve (AUC) value based on the Receiver Operating Characteristics curve. The discrimination slope was calculated as the absolute difference between the mean predicted NTCP value for patients with and without the outcome. The calibration of the model reflects the agreement between observed outcomes and predictions. The calibration slope and intercept were calculated as described by Miller et al. [16]. To evaluate whether the model performance measures based on the observed outcomes differed from their expected values, we used Monte-Carlo to generate the expected distributions and calculated p-values. Finally, a Hosmer-Lemeshow test with 10 groups was performed to evaluate the calibration of the model.

## Results

### Univariate analysis of the training cohort

The training cohort consisted of 427 patients, 77% male and 23% female with a mean age of 62 years. The pretreatment characteristics of the patients are listed in Table 1. Out of 427 patients, 55 (12.9%) were tube feeding dependent at 6 months after completion of treatment. In the univariate analysis, younger age, higher T-classification, higher N-classification, primary tumor site other than the larynx, concomitant chemoradiation, bilateral irradiation, weight loss at baseline and swallowing dysfunction at baseline were significantly associated with TUBE_M6_ (Table 2).

**Table 1:** Pre-treatment charactistics in the training cohort and test cohort.

Variable		Training cohort (n = 427)		Test cohort (n = 183)		P-value
		Number	%	Number	%	
Sex	Male	329	77%	134	73%	p = 0.311
	Female	98	23%	49	27%	
Age	18–65 years	269	63%	117	64%	p = 0.826
	>65 years	158	37%	66	36%	
T-classification	Tis-T1	129	30%	33	18%	p = 0.002
	T2	157	37%	74	40%	
	T3	88	21%	36	20%	
	T4	53	12%	40	22%	
N-classification	N0	291	68%	88	48%	p<0.001
	N1	36	8%	19	10%	
	N2a	9	2%	6	3%	
	N2b	40	9%	16	9%	
	N2c	42	10%	48	26%	
	N3	9	2%	6	3%	
Primary site	Larynx	242	57%	91	50%	p = 0.282
	Oropharynx	103	24%	59	32%	
	Oral cavity	28	7%	10	6%	
	Hypopharynx	33	8%	16	9%	
	Nasopharynx	21	5%	7	4%	
Treatment modality	Conventional radiotherapy	148	35%	12	6%	p<0.001
	Accelerated radiotherapy	204	48%	131	72%	
	Chemoradiation	75	17%	40	22%	
Radiation technique	3D-CRT	379	89%	77	42%	p<0.001
	IMRT	48	11%	106	58%	
Neck irradiation	Primary alone	106	25%	40	22%	p = 0.496
	Primary + ipsilateral neck	33	8%	11	6%	
	Primary + bilateral neck	288	67%	132	72%	
Weigh loss at baseline	No weight loss	320	75%	113	62%	p = 0.002
	Weight loss 1–10%	84	20%	50	27%	
	Weight loss >10%	23	5%	20	11%	
Baseline swallowing	No swallowing problems	338	79%	148	81%	p = 0.601
(grading according to RTOG)	Mild swallowing problems, soft diet	76	18%	32	18%	
	Moderate swallowing problems, liquid diet	13	3%	3	2%	

Abbreviations: 3D-CRT, Three Dimensional Conformal Radiotherapy; IMRT, Intensity-Modulated Radiation Therapy; RTOG, Radiation Therapy Oncology Group.

**Table 2:** Results of the univariate logistic regression analysis with tube feeding dependence at 6 months (TUBE_M6_) as primary endpoint in patients included in the training cohort.

Variable		Univariate analysis		
		Odds ratio	(95% CI)	P-value
Sex	Male	1.00		
	Female	1.61	(0.86–3.00)	p = 0.135
Age	>65 years	1.00		
	18–65 years	2.32	(1.18–4.54)	p = 0.014
T-classification	Tis-T2	1.00		
	T3–T4	10.02	(5.08–19.78)	p<0.001
N-classification	N0	1.00		
	N+	7.67	(4.05–14.50)	p<0.001
Primary site	Larynx	1.00		
	Oral cavity	8.63	(2.92–25.51)	p<0.001
	Oropharynx	8.74	(3.93–19.47)	p<0.001
	Nasopharynx	6.09	(1.70–21.83)	p = 0.006
	Hypopharynx	9.71	(3.52–26.79)	p<0.001
Treatment modality	Conventional radiotherapy	1,00		
	Accelerated radiotherapy	1.77	(0.79–3.99)	p = 0.167
	Chemoradiation	7.72	(3.38–17.67)	p<0.001
Radiation technique	3D-CRT	1.00		
	IMRT	1.67	(0.76–3.67)	p = 0.202
Neck irradiation	Local or unilateral irradiation	1.00		
	Bilateral irradiation	15.45	(3.71–64.39)	p<0.001
Baseline swallowing	Grade 0	1.00		
(grading according to RTOG)	Grade 1–2	3.20	(1.62–6.32)	p = 0.001
Weight loss	No weight loss	1.00		
(baseline)	1–10%	5.66	(2.93–10.94)	p<0.001
	>10%	16.36	(6.42–41.68)	p<0.001

Abbreviations: 3D-CRT, Three Dimensional Conformal Radiotherapy; IMRT, Intensity-Modulated Radiation Therapy; RTOG, Radiation Therapy Oncology Group; CI, Confidence Interval.

### Group-LASSO analysis in training cohort

The LASSO analysis arrived at a multivariable model containing 5 variables with non-zero coefficients: weight loss prior to treatment, T-classification and N-classification, bilateral irradiation of the neck, and treatment modality, including accelerated radiotherapy and chemoradiotherapy (Table 3).

**Table 3:** Results of the LASSO analysis with tube feeding dependence at 6 months (TUBE_M6_) as primary endpoint.

Variable			B	95% CI of B	OR	P-value
T-classification						
	T3–T4 vs. Tis-T2		1.01	(0.79–1.32)	2.75	p<0.001
N-classification						
	N+ vs. N0		0.87	(0.65–1.10)	2.39	p<0.001
Weight loss (baseline)						
	1–10% weight loss vs. no weight loss		0.82	(0.65–0.99)	2.27	p<0.001
	>10% weight loss vs. no weight loss		1.51	(1.19–1.83)	4.53	p<0.001
						
Neck irradiation						
	Bilateral vs. local/unilateral		0.35	(0.06–0.66)	1.42	p = 0.011
						
Treatment modality						
	Chemoradiation vs. conventional fractionation		0.41	(0.16–0.68)	1.51	p = 0.001
	Accelerated fractionation vs. conventional fractionation		0.25	(0.10–0.41)	1.28	p = 0.001
Constant			−3.69	(−4.19–−3.21)		

Abbreviations: OR, Odds Ratio; CI, Confidence Interval; B, model coefficient beta.

In individual cases, the risk of TUBE_M6_ can be estimated using the following equation:
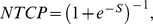
where S = −3.69 + (T-stage * 1.01) + (N-stage * 0.87) + (moderate weight loss * 0.82) + (severe weight loss * 1.51) + (bilateral neck irradiation * 0.35) + (accelerated radiotherapy * 0.25) + (chemoradiotherapy * 0.41)

**Figure 1:**
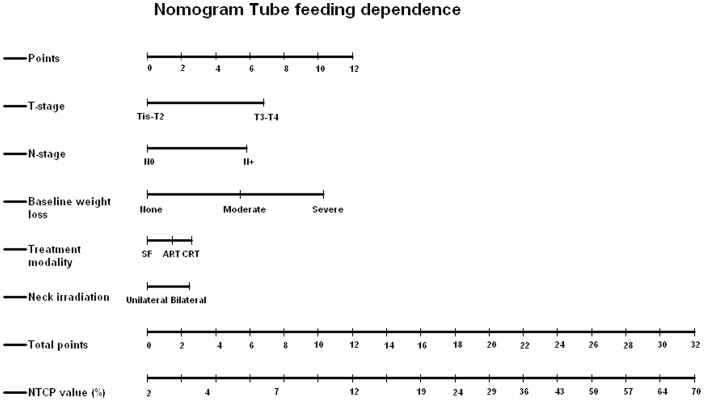
Nomogram for tube feeding dependence to determine normal tissue complication probability (NTCP) values for each individual patient. Abbreviations: SF, conventional radiotherapy; ART, accelerated radiotherapy; CRT, chemoradiation.

The risk of TUBE_M6_ can also be estimated by using the nomogram (Figure 1). In the training cohort, model performance was excellent, with an AUC of 0.86 The discrimination slope had a value of 0.21. The Hosmer-Lemeshow chi square had a value of 9.35 (p-value 0.3) indicating good agreement between expected and observed rates.

### External validation

The test cohort consisted of 183 patients, 73% male and 27% female, with a mean age of 62 years. The training and test cohort differed significantly with regard to T-classification, N-classification, the applied treatment modalities and radiation techniques, and weight loss at baseline (Table 1). Out of 183 patients, 27 (14.8%) were tube feeding dependent at 6 months after completion of treatment. Model performance in the external test cohort was good, with an AUC of 0.82 (Expected: 0.80, 95% CI: 0.72–0.86, p-value: 0.8) and a discrimination slope of 0.20 (Expected: 0.19, 95% CI: 0.13–0.25, p-value 0.6). The calibration graph (Figure S1) illustrates that the observed NTCP-values of TUBE_M6_ in the test cohort are in close proximity of the predicted NTCP-values. The Hosmer-Lemeshow test showed no statistically significant difference between predicted and measured outcomes in the test cohort (Table S1).

### Relationship with tube feeding dependence at subsequent time points

The prevalence of tube feeding dependence was 6.9% (23 of 335 patients at risk) at 12 months (TUBE_M12_), 3.6% (9 of 251 patients at risk) at 18 months (TUBE_M18_) and 4.0% (8 of 200 patients at risk) at 24 months (TUBE_M24_). TUBE_M6_ was very predictive for tube feeding dependence at later time points. The negative predictive values of TUBE_M6_ for TUBE_M12_, TUBE_M18_, TUBE_M24_ were 97.1%, 99.1% and 98.9%, respectively, indicating that almost all patients who were not tube feeding dependent at 6 months remained independent at subsequent time points. The positive predictive values of TUBE_M6_ for TUBE_M12_, TUBE_M18_, TUBE_M24_ were 50.0%, 36.8% and 40.0%, respectively, indicating recovery from tube feeding dependence in more than half of the patients.

## Discussion

The purpose of the current study was to develop and validate a prediction model for tube feeding dependence 6 months after curative (chemo-) radiotherapy in HNC patients. Such a model could be used in clinical practice to predict which patients are at risk for long-term tube feeding dependence, prior to treatment, and would thus be suitable candidates for preventive measures such as swallowing exercises and/or swallowing sparing IMRT.

In the LASSO analysis, five independent prognostic factors for TUBE_M6_ were identified: advanced T-stage (T3–T4), positive N-stage, weight loss at baseline, bilateral irradiation of the neck, and treatment modality. Model performance in both the training cohort and the test cohort from another hospital was good to excellent, which confirms the generalization ability of the model.

A prediction model as presented in this study is increasingly desirable due to the more aggressive treatment regimens that are being used in HNC, including altered fractionation schedules for radiotherapy, concomitant (chemo-) radiotherapy or both. These intensive cancer treatments have improved loco-regional control and overall survival [17]–[19] but at the expense of an increase in radiation-induced side effects [20] in particular long-term swallowing dysfunction [21].

Prophylactic feeding tube placement is standard practice in many institutions to avoid treatment interruptions and unplanned hospitalizations because of compromised nutritional intake and/or dehydration [22]; adequate nutrition has been shown to improve tolerance and response rates to (chemo-) radiotherapy [23].

However, others have shown that pretreatment feeding tube placement may lead to increased long-term swallowing dysfunction, longer feeding tube duration and the need for pharyngo-esophageal dilatation [24]. In addition, long-term feeding tube dependence may significantly reduce quality of life after treatment for HNC [22], [25]. Terrell *et al.* [26] showed that feeding tube dependence was the strongest clinical predictor of negative effects on health-related quality of life relative to other medical co-morbidities. It was associated with significantly lower scores on 10 out of 12 collective domains in the Medical Outcomes Study Short Form 36-item Health Survey and HNC quality-of-life instruments. As already mentioned, at both institutions, prophylactic PEG tube placement was standard of care in all patients treated with curative concomitant chemoradiation and patients were instructed to refrain from using the PEG-tube. In patients with significant weight loss (>5% weight loss in 1 month or >10% in 6 months or BMI <18.5 kg/m^2^) and/or low nutritional intake (less than half of daily requirements for energy, proteins or fluids) and/or severe swallowing dysfunction prior to treatment, PEG tubes were placed prior to treatment. However, these patients were excluded from the analysis.

Chemoradiotherapy was a prognostic factor in the LASSO analysis. It should be stressed that given the fact that all patients receiving concomitant chemoradiation received prophylactic PEG tube placement, the Odds ratio of 1.51 should be considered the results of this preset combination and that no conclusions can be drawn with regard to these two factors separately. However, given this Odds ratio of 1.51 and the Odds ratio of 1.28 found for accelerated radiotherapy (without prophylactic PEG-tube placement), we believe that the contribution of prophylactic PEG tube feeding is probably limited or absent.

The training cohort and test cohort were from two different hospitals with different chemotherapy regimens in each hospital. In the training cohort, cisplatinum was used while in the test cohort 5-FU in combination with carboplatin was used. The model performance in both cohorts was comparable, which indirectly confirms that there will probably be no major difference between the two chemotherapy regimens.

Given that both advanced T-stage (larger tumors) and N-stage and bilateral irradiation of the neck were prognostic factors for feeding tube dependence at 6 months after (chemo-) radiotherapy, it can be hypothesized that the risk of tube feeding dependence is related to the radiation dose distribution in the anatomical structures involved in swallowing, such as the pharyngeal constrictor muscles. A number of authors indeed showed a dose-volume-effect relationships of anatomical structures involved in swallowing and swallowing dysfunction after (chemo-) radiotherapy, such as the pharyngeal constrictor muscles [8], [27]–[29].

Accelerated radiotherapy was also an independent prognostic factor for tube feeding dependence. These results are in line with those presented by Overgaard *et al.* who showed that accelerated radiotherapy lead to more frequent and longer persisting confluent mucositis than the conventionally treated group and consequently long-term dysphagia [30]. Another more recent updated study, however, showed that accelerated RT does increase acute but not late morbidity, including dysphagia [31].

In the current study, baseline weight loss was also an independent prognostic factor for tube feeding dependence. This is in accordance with a previous report [1] in which baseline weight loss was shown to be an independent prognostic factor for grade 2–4 RTOG swallowing dysfunction at 6 months after treatment. This suggests that a relatively high percentage of baseline swallowing dysfunction is not recognized by radiation oncologists and that swallowing dysfunction is therefore often underreported, even in the case of prospective assessment of toxicity.

From clinical practice we know that the reasons for feeding tube placement are not solely related to swallowing problems, but may be multifactorial. Nausea due to chemotherapy treatment, changes in taste or saliva production and other factors may also necessitate feeding tube placement. We did not directly take these factors into account in this analysis since it was not possible to obtain information about this for each individual patient. This is something, however, that may have influenced feeding tube placement and use in this patient group.

As the main purpose of the current analysis was to develop and validate a multivariable prediction model that can be used to select patients prior to treatment (i.e. during the preparation phase of radiotherapy) for preventive measures, we did not take into account candidate variables related to dose distributions in swallowing organs at risk. This, however, will be investigated in future research.

Our results show that TUBE_M6_ after treatment is predictive for tube feeding dependence at later time points up to 24 months after completion of radiotherapy. This is also in accordance with previous reports [1], which showed that swallowing dysfunction at 6 months after curative (chemo-) radiation is highly predictive for swallowing dysfunction at subsequent time points up to several years after treatment.

## Conclusion

The present study is the first to provide an externally validated prediction model for tube feeding dependence after (chemo-) radiation in a population-based cohort of patients with HNC. This model enables clinicians to select patients that have not yet started treatment, based on pretreatment characteristics, who are at greatest risk for tube feeding dependence after treatment, and to implement preventive strategies for them.

## Supporting Information

Figure S1 Calibration plots for the predictive model for tube feeding dependence at 6 months (TUBE_M6_) at internal validation (A) and external validation (B).
(TIF)

Table S1 Performance of the prediction model for TUBEM6. Abbreviations: AUC, Area Under Curve; H-L, Hosmer-Lemeshow.
(DOC)
