# Supplementary figures and images for: Development and Validation of a Prediction Model for Tube Feeding Dependence after Curative (Chemo-) Radiation in Head and Neck Cancer
Source: PLoS One. 2014 Apr 15;9(4):e94879. doi: 10.1371/journal.pone.0094879 (PMC3988098; doi:10.1371/journal.pone.0094879)

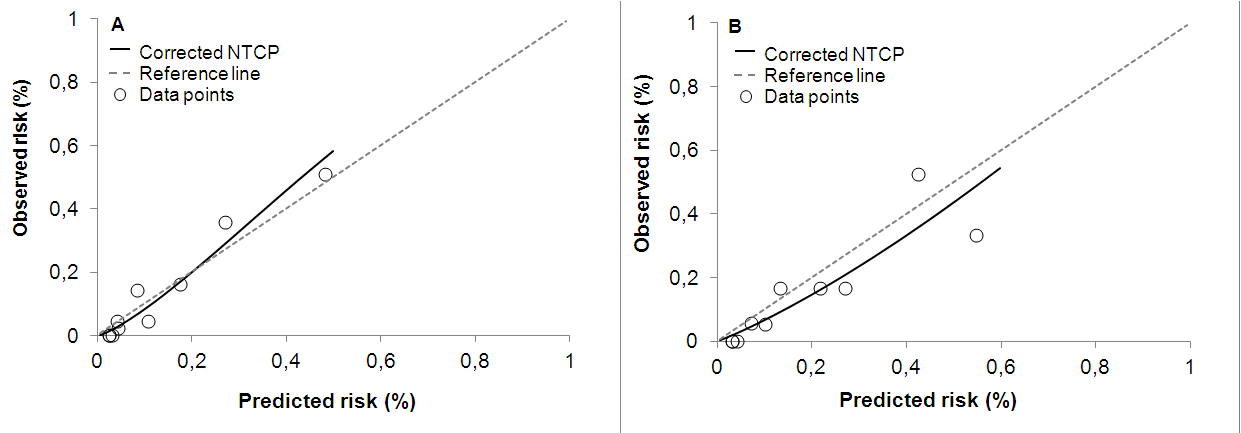

Supplement: Figure S1 — Calibration plots for the predictive model for tube feeding dependence at 6 months (TUBEM6) at internal validation (A) and external validation (B). (TIF) [file pone.0094879.s001.tif]
