# Supplementary material for: Development and Validation of a Prediction Model for Tube Feeding Dependence after Curative (Chemo-) Radiation in Head and Neck Cancer
Source: PLoS One. 2014 Apr 15;9(4):e94879. doi: 10.1371/journal.pone.0094879 (PMC3988098; doi:10.1371/journal.pone.0094879)
Supplement: Table S1 — Performance of the prediction model for TUBEM6. Abbreviations: AUC, Area Under Curve; H-L, Hosmer-Lemeshow. (DOC) [file pone.0094879.s002.doc]

| **Table S1: Performance of the prediction model for TUBE_M6_.** | | | | | | |
| --- | --- | --- | --- | --- | --- | --- |
| **Performance measure** | **Internal validation** |  |  | **External validation** |  |  |
|  | **Actual** | **p-value** |  | **Actual** | **p-value** |  |
| **Discrimination** |  |  |  |  |  |  |
| *Discrimination slope* | 0.21 | 0.90 |  | 0.20 | 0.60 |  |
| *AUC* | 0.86 | 0.98 |  | 0.82 | 0.80 |  |
| **Calibration** |  |  |  |  |  |  |
| *Calibration intercept* | 0.33 |  |  | -0.26 |  |  |
| *Calibration slope* | 1.24 |  |  | 1.09 |  |  |
| *H-L test* | x^2^ = 9.35 | 0.30 |  | x^2^ = 8.12 | 0.50 |  |
| **Explained variance** |  |  |  |  |  |  |
| *Nagelkerke R2* | 0.35 | 0.96 |  | 0.26 | 0.50 |  |
|  |  |  |  |  |  |  |
| Abbreviations: AUC, Area Under Curve; H-L, Hosmer-Lemeshow. | | |  |  |  |  |
